# Crystal structure and conformational flexibility of the unligated FK506-binding protein FKBP12.6

**DOI:** 10.1107/S1399004713032112

**Published:** 2014-02-15

**Authors:** Hui Chen, Sourajit M. Mustafi, David M. LeMaster, Zhong Li, Annie Héroux, Hongmin Li, Griselda Hernández

**Affiliations:** a Wadsworth Center, New York State Department of Health, Empire State Plaza, Albany, NY 12201, USA; b Department of Biomedical Sciences, School of Public Health, University at Albany – SUNY, Empire State Plaza, Albany, NY 12201, USA; c Department of Biology, Brookhaven National Laboratory, Upton, NY 11973, USA

**Keywords:** FKBP12.6, FK506-binding proteins

## Abstract

The primary known physiological function of FKBP12.6 involves its role in regulating the RyR2 isoform of ryanodine receptor Ca^2+^ channels in cardiac muscle, pancreatic β islets and the central nervous system. With only a single previously reported X-ray structure of FKBP12.6, bound to the immunosuppressant rapamycin, structural inferences for this protein have been drawn from the more extensive studies of the homologous FKBP12. X-ray structures at 1.70 and 1.90 Å resolution from *P*2_1_ and *P*3_1_21 crystal forms are reported for an unligated cysteine-free variant of FKBP12.6 which exhibit a notable diversity of conformations. In one monomer from the *P*3_1_21 crystal form, the aromatic ring of Phe59 at the base of the active site is rotated perpendicular to its typical orientation, generating a steric conflict for the immunosuppressant-binding mode. The peptide unit linking Gly89 and Val90 at the tip of the protein-recognition ‘80s loop’ is flipped in the *P*2_1_ crystal form. Unlike the >30 reported FKBP12 structures, the backbone conformation of this loop closely follows that of the first FKBP domain of FKBP51. The NMR resonances for 21 backbone amides of FKBP12.6 are doubled, corresponding to a slow conformational transition centered near the tip of the 80s loop, as recently reported for 31 amides of FKBP12. The comparative absence of doubling for residues along the opposite face of the active-site pocket in FKBP12.6 may in part reflect attenuated structural coupling owing to increased conformational plasticity around the Phe59 ring.

## Abstract 2

Two crystal forms of unligated FKBP12.6 exhibit multiple conformations in the active site and in the 80s loop, the primary site for known protein-recognition interactions. The previously unreported NMR backbone assignment of FKBP12.6 revealed extensive doubling of amide resonances, which reflects a slow conformational transition centered in the 80s loop.

## 1. Introduction   

The human genome encodes two homologous 12 kDa FK506-binding proteins, FKBP12 and FKBP12.6, which differ at 18 residue positions (Supplementary Fig. S1^1^). Each of these proteins can bind the immunosuppressant FK506 to form a ternary complex with the protein phosphatase calcineurin which, by inhibiting the activity of this enzyme, blocks a key T-­cell activation pathway involved in tissue transplant rejection (Liu *et al.*, 1991 ▶). Similarly, both FKBP12 and FKBP12.6 can bind the immunosuppressant rapamycin to form an inhibitory ternary complex with the protein kinase mTOR (mammalian target of rapamycin), which plays a major role in regulating cell growth and cancer progression (Heitman *et al.*, 1991 ▶). In both of these pathways, FKBP12 is believed to be the primary mediator of FK506-induced and rapamycin-induced immunosuppression. As a result, a large number of structural and biochemical studies have been reported for FKBP12, as illustrated by the deposition of over 30 X-ray structures of the wild type and mutational variants of the human protein both unligated and either bound to small-molecule inhibitors or in physiological protein–protein complexes. In marked contrast, only a single X-ray structure of FKBP12.6 has been deposited in the Protein Data Bank, in which the protein is bound to rapamycin (Deivanayagam *et al.*, 2000 ▶).

The exclusive role of FKBP12 in mediating these immunosuppressive effects has recently been called into question by the demonstration that not only FKBP12.6 but also several of the larger FKBP domain-containing proteins inhibit mTOR catalytic activity *in vitro* at similar rapamycin concentrations as for FKBP12, and both FKBP51 and FKBP52 can functionally replace FKBP12 in a cellular model system of mTOR inhibition (März *et al.*, 2013 ▶). Furthermore, siRNA studies indicate that FKBP12 contributes ∼60% of the FK506-mediated inhibition of calcineurin, while the rest of the inhibitory effect is contributed nearly equally by FKBP12.6 and FKBP51 (Weiwad *et al.*, 2006 ▶).

Despite the substantial pharmacological relevance, the interaction of human FKBP12 and FKBP12.6 with the bacterial macrolides FK506 and rapamycin appears to be nonphysiological. Over two decades of research have not revealed an endogenous small-molecule ligand that mediates protein–protein interactions for the FKBP domains (Galat, 2013 ▶). In addition to FKBP12, FKBP12.6 and the more distantly related FKBP13, there are 19 other FKBP domains in the human genome that exist as one or more modules within larger protein sequences (Galat, 2008 ▶). Some, but not all, of these FKBP domains catalyze the *cis*–*trans* prolyl isomerization reaction. The relative significance of this catalytic activity for the biological function of FKBP12 and FKBP12.6 remains an open question, although the prolyl isomerization activity of FKBP12 has been proposed to contribute to the interactions of this protein with the misfolded forms of α-synuclein (Gerard *et al.*, 2010 ▶), tau (Sugata *et al.*, 2009 ▶) and amyloid precursor proteins (Liu *et al.*, 2006 ▶). To date, all of the functionally characterized roles of FKBP12 and FKBP12.6 as well as of the human FKBP-domain proteins specifically discussed below involve the formation of specific protein–protein complexes, generally with proteins participating in various signaling pathways.

Both FKBP12 and FKBP12.6 are involved in the regulation of ryanodine receptor Ca^2+^ channels. FKBP12 preferentially binds to the RyR1 isoform found in skeletal muscle (Timerman *et al.*, 1993 ▶), while FKBP12.6 preferentially binds to the RyR2 isoform found in cardiac muscle (Lam *et al.*, 1995 ▶; Timerman *et al.*, 1996 ▶). Most subsequent studies have been interpreted in terms of distinct regulation of skeletal muscle RyR1 by FKBP12 and cardiac RyR2 regulation by FKBP12.6. However, a number of studies have pointed to a more complex pattern of interactions (Galfré *et al.*, 2012 ▶; Maruyama *et al.*, 2011 ▶). Protein kinase A phosphorylation of the cardiac RyR during myocardial infarction has been argued to lead to dissociation of FKBP12.6 (Wehrens *et al.*, 2005 ▶), and drugs designed to prevent this dissociation have been proposed to inhibit heart failure progression in mice (Shan *et al.*, 2010 ▶), although this approach remains controversial (Bers, 2012 ▶; Eschenhagen, 2010 ▶).

The RyR2 isoform is predominant in pancreatic islet cells (Okamoto & Takasawa, 2002 ▶), and FKBP12.6 regulation of these Ca^2+^ channels is indicated by FK506-induced calcium release (Noguchi *et al.*, 1997 ▶). Although the blood glucose and plasma insulin concentrations for FKBP12.6^−/−^ mice were normal in both random and fasted states, insulin secretion in response to glucose tolerance stress in these FKBP12.6-deficient mice was severely depressed relative to their FKBP12.6^+/+^ littermates (Noguchi *et al.*, 2008 ▶). RyR2 channels are also abundant in the central nervous system. Antisense oligonucleotide knockdown experiments in mice showed impairment of memory processes, in contrast to analogous experiments for the RyR1 channels (Galeotti *et al.*, 2008 ▶). Recent evidence suggests that release of FKBP12.6 from neuronal RyR2 channels may play a crucial role in stress-induced cognitive dysfunctions such as post-traumatic stress disorder (Liu *et al.*, 2012 ▶). Analogous to the earlier analysis of heart failure, drugs that inhibit the dissociation of FKBP12.6 provide a potential avenue for therapy (Liu *et al.*, 2012 ▶).

The range of clinical pathologies in which FKBP12.6-mediated signaling appears to play a significant role provides impetus for structure-based rational drug design to modulate the biological activities of this protein. Developing adequate pharmacological selectivity is not only crucial with respect to its close homolog FKBP12 but also for the other FKBP domain-containing proteins, such as the actively studied FKBP38, FKBP51 and FKBP52 (Supplementary Fig. S1). A subset of the diverse physiological functions for this family of proteins is illustrated by FKBP domain-mediated binding of FKBP38 to the anti-apoptosis protein Bcl-2 (Maestre-Martínez *et al.*, 2011 ▶), which targets it to the mitochondrial membrane (Edlich *et al.*, 2005 ▶; Shirane & Nakayama, 2003 ▶). FKBP38 further facilitates chemoresistance by binding to the caspase cleavage site of Bcl-2, thus interfering with its degradation (Choi *et al.*, 2010 ▶). The first FKBP domain of both FKBP51 and FKBP52 serves as the primary regulatory domain for the interactions within the glucocorticoid receptor complex, in which FKBP52 selectively potentiates hormone-dependent gene activation while FKBP51 antagonizes this potentiation (Riggs *et al.*, 2003 ▶). The complementary antagonism between FKBP51 and FKBP52 is further illustrated by the role of this FKBP domain in the critical balancing of the phosphorylation state of the tau protein in activating (FKBP51; Jinwal *et al.*, 2010 ▶) or inhibiting (FKBP52; Chambraud *et al.*, 2010 ▶) the ability of tau to promote microtubule polymerization.

To provide a broader base for selectivity design among the FKBP proteins, we report structures of the unligated form of a cysteine-free variant of FKBP12.6 in two different crystal space groups that exhibit substantial conformational heterogeneity, as well as NMR measurements to further characterize the plasticity of this protein.

## 2. Methods   

### 2.1. Protein preparation   

Genes for the wild type as well as the C22V+C76I and H87V variants of human FK506-binding protein FKBP12.6 were chemically synthesized (GenScript) from the wild-type gene sequence, with codon optimization for expression in *Escherichia coli*. The genes were cloned into the expression vector pET-11a and then transformed into the BL21(DE3) strain (Novagen) for expression.

The protein expression and purification procedure for the homologous FKBP12 protein (Hernández *et al.*, 2009 ▶) was followed through to the Sephadex 50 gel-filtration step with 1 m*M* dithiothreitol added to all buffers used in the wild-type FKBP12.6 purification. Eluted fractions from the gel-filtration column were loaded onto a 5 ml column of SP Sepharose equilibrated in 50 m*M* Tris–acetate pH 8.0 buffer. After washing with this buffer, 0.1 *M* sodium chloride was added to the buffer and the protein sample was eluted from the column.

All isotopically labeled samples were prepared *via* protein expression in minimal medium containing 0.1% ^15^NH_4_Cl as a nitrogen source, as described previously (Hernández & LeMaster, 2001 ▶). For U-^2^H,^13^C,^15^N-enriched samples, 0.2% U-^2^H,^13^C glucose (Cambridge Isotopes) was substituted for the unlabeled glucose used for preparing the U-^15^N samples. The U-^2^H,^15^N-enriched samples were prepared using U-^2^H glycerol (Cambridge Isotopes) as a carbon source in a ^2^H_2_O-­containing minimal medium as described previously (Hernández & LeMaster, 2001 ▶).

For the protein samples expressed in ^2^H_2_O medium, solid Tris base was added to a solution of the purified protein to obtain a pH value above 9 and the samples were incubated at 25°C for at least 3 h and then neutralized with solid monobasic sodium phosphate. For wild-type FKBP12.6, 1 m*M* tris(2-carboxyethyl)phosphine was added for this exchange step. All protein samples were concentrated *via* centrifugal ultrafiltration and then equilibrated into a pH 7.00 buffer containing 25 m*M* sodium phosphate [and 2 m*M* dithiothreitol and 2 m*M* tris(2-carboxyethyl)phosphine for the wild-type protein] by a series of centrifugal concentration steps. For the crystallization trials, the protein sample was neutralized and then equilibrated into 5 m*M* sodium chloride and concentrated by centrifugal ultrafiltration.

### 2.2. Crystallization and structure determination   

Crystals of the C22V+C76I variant of the FKBP12.6 protein were grown at room temperature in hanging drops by mixing 2 µl protein solution at 45 mg ml^−1^ concentration with an equal volume of reservoir solution consisting of either 2.0 *M* malonic acid pH 7.0, 4% 2-propanol (condition 1) or 62% ammonium sulfate, 0.1 *M* HEPES pH 7.5, 4% 2-propanol (condition 2).

The crystals from condition 1 belonged to space group *P*2_1_, with unit-cell parameters *a* = 39.06, *b* = 49.35, *c* = 50.98 Å, β = 101.82° (Table 1 ▶). There are two molecules per asymmetric unit. Prior to data collection, crystals were gradually transferred to reservoir solution containing higher concentrations of malonic acid up to 4.0 *M* at 0.5 *M* per step, and then flash-cooled under a nitrogen stream at 100 K and stored in liquid nitrogen. Diffraction data were collected at 100 K using an R-­AXIS IV^++^ detector and an in-house Rigaku microfocus MicroMax-007 X-ray generator. Diffraction data were processed and scaled using *CrystalClear* 1.3.6 (Rigaku Corp.).

The crystals from condition 2 belonged to space group *P*3_1_21, with unit-cell parameters *a* = *b* = 52.816, *c* = 152.949 Å, γ = 120° (Table 1 ▶). There are two molecules per asymmetric unit. Prior to data collection, crystals were gradually transferred to reservoir solution containing higher concentrations of ammonium sulfate up to 80% at 5% increment per step, and then flash-cooled under a nitrogen stream at 100 K and stored in liquid nitrogen. Diffraction data were collected at 100 K on beamline X25 at the National Synchrotron Light Source, Brookhaven National Laboratory. Diffraction data were processed and scaled using *HKL*-2000 (Otwinowski & Minor, 1997 ▶).

Using the crystal structure of FKBP12.6 in complex with rapamycin (PDB entry 1c9h; Deivanayagam *et al.*, 2000 ▶) as a search model, clear solutions were found with the *Phaser* molecular-replacement program within the *PHENIX* suite (Adams *et al.*, 2010 ▶). Structural refinement was carried out using *PHENIX*. Model rebuilding was carried out using *Coot* (Emsley *et al.*, 2010 ▶). Figures of crystallographic structures were generated using the *Chimera* software (Pettersen *et al.*, 2004 ▶).

### 2.3. NMR spectroscopy   

In contrast to the more stable FKBP12, at pH 6 and 25°C the ^1^H–^15^N two-dimensional correlation spectra of FKBP12.6 accumulate additional cross-peaks with a limited dispersion in ^1^H chemical shift, as is typical of disordered structures. When the pH is raised to 7 in the presence of thiol reducing agents, samples of the wild-type FKBP12.6 were suitably stable for the time periods needed for multidimensional resonance-assignment experiments. The cysteine-free variant of FKBP12.6 was used for the *zz*-exchange measurements at elevated temperatures. NMR assignment data were collected on a Bruker Avance III 600 MHz spectrometer and a Bruker Avance II 800 MHz spectrometer at 25°C. Backbone resonance assignments were carried out using standard HNCO (Kay *et al.*, 1994 ▶), HN(CA)CO (Kay *et al.*, 1994 ▶), HNCACB (Muhandiram & Kay, 1994 ▶) and HN(CO)CACB (Yamazaki *et al.*, 1994 ▶) experiments. The *FELIX* software (Felix NMR) was used for NMR data processing. The backbone resonance assignments for the unligated wild-type human FKBP12.6 have been deposited in the Biological Magnetic Resonance Bank (BMRB accession Nos. 19323 and 19324 for the major and minor slow-exchange states, respectively).

## 3. Results and discussion   

### 3.1. Overall protein structure   

Relative to FKBP12, our initial NMR studies indicated a reduced sample stability of wild-type FKBP12.6 at temperatures above 25°C and pH values below 7. We (Mustafi *et al.*, 2013 ▶) recently reported the use of a C22V variant in temperature-dependent studies of FKBP12, in which the evolutionarily conservative valine substitution eliminates the complications of cysteine reactivity that occur at higher pH and/or temperature conditions. Substitution of valine at residue 22 into human FKBP12 results in a minimal perturbation in the crystal structure around this site, with the only appreciable shift in heavy-atom position being for Leu103 C^δ1^, which moves 0.6 Å away from the newly introduced Val22 C^γ1^ methyl group (Mustafi *et al.*, 2013 ▶). NMR relaxation analysis of both the wild type and the C22V variant of FKBP12 indicated closely similar backbone conformational dynamics in both the picosecond-to-nanosecond and the microsecond-to-millisecond timeframes (Mustafi *et al.*, 2013 ▶). In addition to Cys22, human FKBP12.6 contains a second cysteine at residue 76. Human FKBP12 contains an isoleucine residue at position 76, as do the FKBP12 proteins from lower eukaryotes such as yeast and *Caenorhabditis elegans* (Somarelli & Herrera, 2007 ▶), indicating a functional tolerance to this substitution (FKBP12 and FKBP12.6 diverged during the evolution of fish).

A survey of crystallization conditions for the cysteine-free variant of FKBP12.6 yielded two distinct crystal forms, one belonging to space group *P*2_1_, yielding a data set at 1.70 Å resolution, and one belonging to space group *P*3_1_21, yielding a data set at 1.90 Å resolution (Table 1 ▶). Similar to the observations made for the C22V variant of FKBP12, for both crystal forms of the C22V+C76I variant of FKBP12.6 the interactions surrounding residue 22 are highly similar to those for the 1.7 Å resolution wild-type rapamycin-inhibited form of the protein (Deivanayagam *et al.*, 2000 ▶). As illustrated by the *P*3_1_21 crystal form of the unligated protein (Supplementary Fig. S2), the interactions surrounding residue 76 are largely similar to those for the rapamycin-inhibited wild-type FKBP12.6, with the maximum backbone perturbation occurring at Val80 C^α^, which is displaced by less than 0.7 Å owing to steric inter­action with C^γ2^ of the substituted isoleucine side chain.

The two non-equivalent monomers in the asymmetric unit of the *P*3_1_21 crystal form differ from each other by only 0.35 Å r.m.s.d. for all of the backbone heavy atoms. Each of these monomers differs from the 0.92 Å resolution crystal structure of unligated FKBP12 (PDB entry 2ppn; Szep *et al.*, 2009 ▶) by a backbone r.m.s.d. of 0.50 Å. In contrast, both of these unligated FKBP12.6 monomers differ from the rapamycin-inhibited FKBP12.6 (Deivanayagam *et al.*, 2000 ▶) by an appreciably larger backbone r.m.s.d. of 0.81 Å. These larger differences primarily arise from the transition between a type I and a type II turn for residues Gln31–Lys34 in the unligated and rapamycin-bound crystals, respectively. Similarly, the previously reported crystal structure of rapamycin-inhibited FKBP12 (Van Duyne *et al.*, 1993 ▶) yielded a type II turn for this segment, in contrast to all previously reported crystal structures of FK506-inhibited and unligated FKBP12. Despite the substitution of the doubly charged Glu31–Asp32 dipeptide of FKBP12 with the neutral Gln31–Asn32 dipeptide of FKBP12.6, the two rapamycin-inhibited proteins crystallized in closely similar crystal forms. The earlier crystallographic study of rapamycin-inhibited FKBP12 proposed that lattice interactions account for the change in conformation of the turn, and this interpretation was supported by subsequent crystal structures of the ternary complex containing FKBP12, rapamycin and the FKBP-rapamycin binding domain of mTOR that contain the more commonly observed type I conformation for this turn (Choi *et al.*, 1996 ▶).

The conformational plasticity of this turn segment gains potential physiological relevance from the observation that introducing the neutral Gln31–Asn32 dipeptide and active-site Phe59 from the FKBP12.6 sequence in place of the doubly charged Glu31–Asp32 dipeptide and Trp59 of FKBP12 generates a variant of FKBP12 with a 600-fold enhanced binding selectivity for the cardiac ryanodine receptor, equivalent to that of wild-type FKBP12.6 (Xin *et al.*, 1999 ▶). Each of the three residues was found to contribute a similar proportion to the overall selectivity enhancement.

### 3.2. Conformational plasticity in the active site of unligated FKBP12.6   

As noted in the crystallographic analysis of rapamycin-inhibited FKBP12.6 (Deivanayagam *et al.*, 2000 ▶), the center of the Phe59 ring is superimposed upon the center of the larger indole ring of Trp59 in the homologous FKBP12 X-ray structure. In both cases, the side chain of this aromatic residue extends away from the α-helix to form the floor of the active-site pocket. Given the shorter phenylalanine side chain in FKBP12.6, this superpositioning of the active-site aromatic ring positions is achieved by a 1 Å shift in the α-helix towards the center of the β-sheet. Mutational analysis of the cardiac ryanodine receptor-selective variant of FKBP12 discussed above (Xin *et al.*, 1999 ▶) stimulated a subsequent crystallo­graphic analysis of the W59F variant of FKBP12, which concluded that this substitution is sufficient to enable a shift of the α-helix so that the positioning of the aromatic ring at the base of the active-site pocket could be preserved (Fulton *et al.*, 2003 ▶).

**Figure 1:**
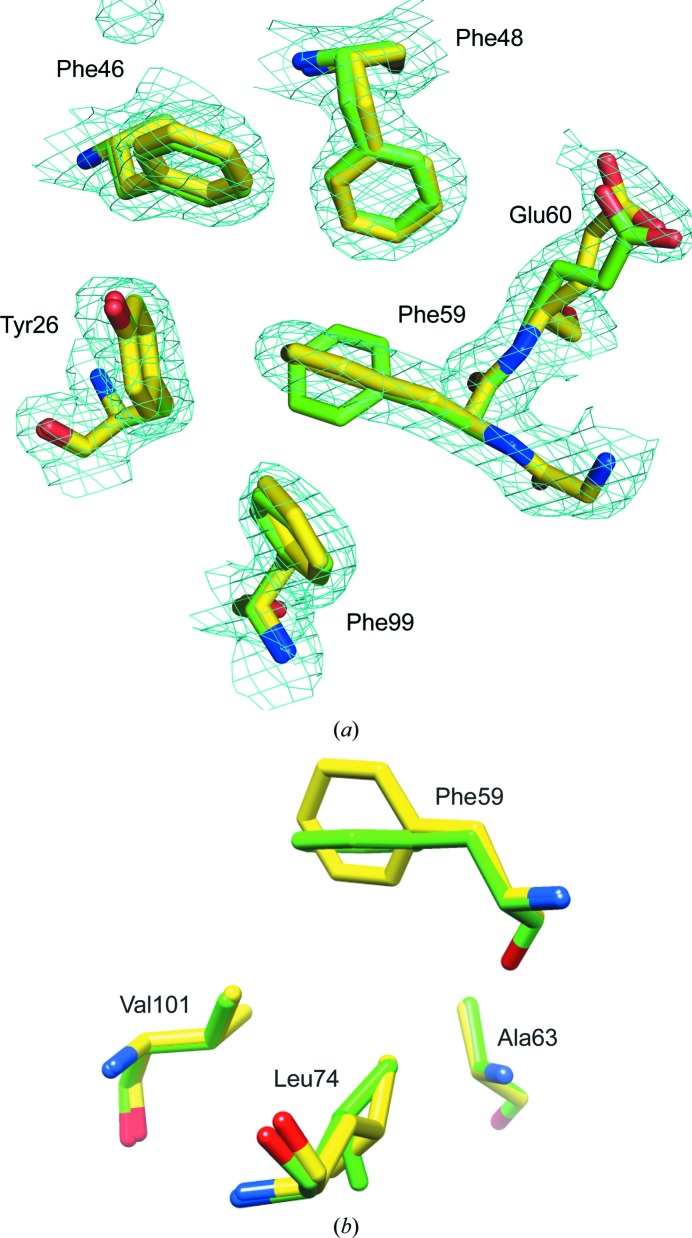
Superposition of the region surrounding the aromatic ring of Phe59 from the two non-equivalent monomers in the 1.90 Å resolution structure of the *P*3_1_21 crystal form of the cysteine-free variant of FKBP12.6. The C atoms of the molecule *A* structure are shown in yellow, while those of molecule *B* are indicated in green. In (*a*), the electron-density grid (2*mF*_o_ − *DF*_c_ at a contour level of 0.0114 e Å^−3^ = 1σ) for molecule *A* is also illustrated, indicating that the aromatic ring of Phe59 from this molecule is oriented perpendicular to that from molecule *B*. In the latter case, the plane of the ring forms the base of the active-site cleft, as seen in previously reported crystal structures of FKBP proteins. Among the residues lying beneath the ring of Phe59 (*b*), only Val101 C^γ2^ lies within van der Waals contact with either the C^δ^ or C^∊^ atoms of the Phe59 ring for the two orientations in the *P*3_1_21 crystal form. The side chains of Ala63 and Leu74 C^δ^ are approximately 1 Å beyond van der Waals contact.

The present crystallographic analysis of unligated FKBP12.6 provides further insight into the conformational plasticity of the active site. Despite the close alignment of the backbone heavy atoms for the two non-equivalent monomers in the asymmetric unit of the *P*3_1_21 crystal form (0.35 Å r.m.s.d.), the orientation of the aromatic ring of Phe59 at the base of the active-site cleft is strikingly different. In molecule *B* the phenylalanine ring superimposes upon that seen in the rapamycin-inhibited FKBP12.6, which in turn is closely similar to the orientation of the Trp59 ring in the numerous X-ray structures of the wild-type FKBP12 protein. However, in molecule *A* the electron-density map clearly indicates a perpendicular orientation of the Phe59 ring (Fig. 1 ▶ *a*). The rest of the active-site cavity is largely unperturbed by the reorientation of the Phe59 ring, as illustrated for the aromatic rings of Tyr26, Phe46, Phe48 and Phe99 which line the walls of this cavity. Similarly, the reorientation of the Phe59 side chain is accompanied by minimal perturbations in the conformation of the residues that lie beneath the base of the active-site pocket (Fig. 1 ▶ *b*). Only Val101 C^γ2^ lies within van der Waals contact with either the C^δ^ or C^∊^ atoms of Phe59 (3.8 Å for the parallel orientation of molecule *B* and 3.6 Å for the perpendicular orientation of molecule *A*). Ala63 C^β^ and Leu74 C^δ^ are separated from these ring C atoms by at least 4.8 and 4.6 Å, respectively.

**Figure 2:**
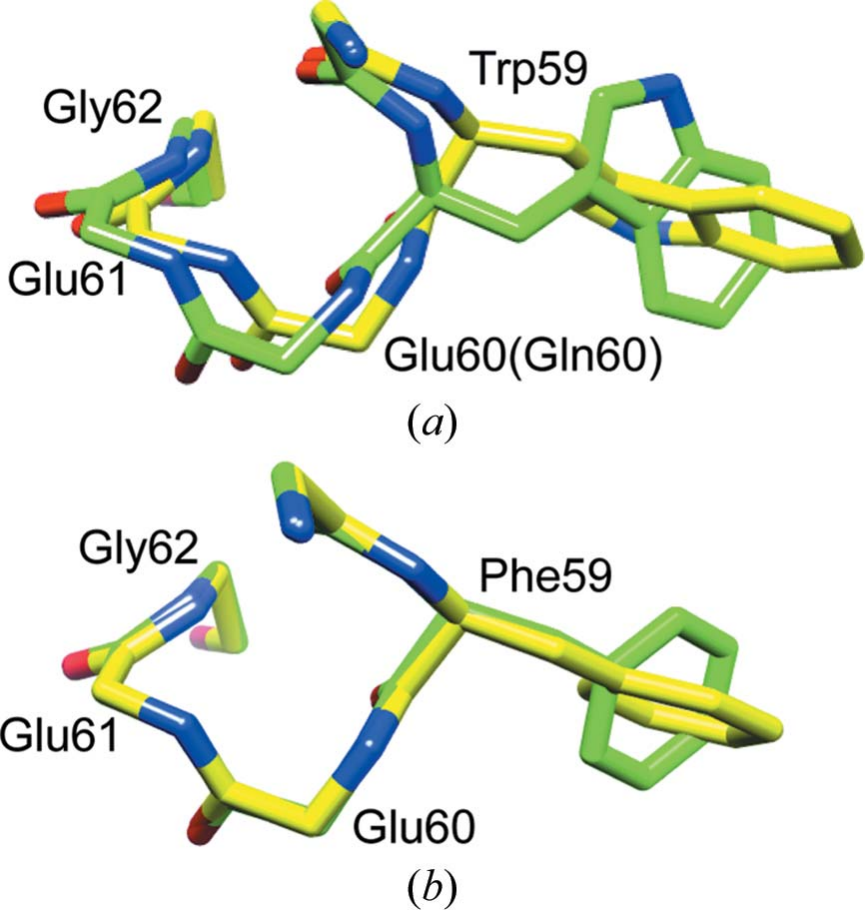
Backbone conformation in the active site of wild-type and E60Q FKBP12 and of the crystallographically non-equivalent monomers of FKBP12.6 in the *P*3_1_21 crystal form. (*a*) shows the shift in backbone conformation and the reorientation of the Trp59 side chain that results from the altered hydrogen-bonding interactions of the side chain of residue 60 (Szep *et al.*, 2009 ▶). A similar ∼90° rotation of the side-chain χ_2_ dihedral angle for Phe59 occurs between the two non-equivalent monomers in the *P*3_1_21 crystal form of the cysteine-free variant of FKBP12.6, but without any corresponding alteration in the conformation of the backbone (*b*).

This perpendicular reorientation of the Phe59 aromatic ring is strongly reminiscent of the reorientation of the Trp59 ring that was recently reported for the E60Q variant of FKBP12 (PDB entry 2ppp; Szep *et al.*, 2009 ▶). In both the 0.92 Å resolution structure of wild-type unligated FKBP12 and the present structure of unligated FKBP12.6, the Glu60 side chain (Fig. 1 ▶ *a*) extends towards the backbone of the 50s loop to coordinate a solvent-inaccessible water molecule that in turn bonds to the amide H atoms of Lys52 and Glu54 as well as the carbonyl O atom of Met49 (Szep *et al.*, 2009 ▶). The introduction of glutamine at position 60 of FKBP12 results in the flipping of the peptide unit that links Lys52 and Gln53 and a shift in the coordination of the buried water molecule in the 2ppp structure. An attendant reorientation of the α-helix results in a 1.5 Å shift in the C^α^ position of Gln60 with respect to the Glu60 residue in the wild-type FKBP12 structure (Fig. 2 ▶ *a*). These authors concluded that this shifting of the backbone causes a severe steric collision of the indole ring with the phenyl ring of Phe99 that is relieved by the reorientation of the Trp59 side chain (Szep *et al.*, 2009 ▶). In this regard, it should be noted that NMR studies also indicate that the indole H^N^ of Trp59 is tightly pressed against the ring of Phe99 in solution, giving rise to a strongly upfield-shifted ^1^H resonance, as demonstrated previously for the homologous interaction in the first FKBP domain of FKBP52 (Craescu *et al.*, 1996 ▶). The reoriented Trp59 side chain of the E60Q variant of FKBP12 serves to partially occlude the active-site space that is normally occupied by the FK506 ligand.

The reoriented aromatic ring of Phe59 in the presently reported structure of FKBP12.6 likewise partially occludes the active-site binding pocket. However, in contrast to the E60Q variant of FKBP12, the reorientation of the Phe59 ring is not accompanied by an alteration in the local backbone conformation (Fig. 2 ▶ *b*). Furthermore, the positions of the aromatic rings of Phe48 and Phe99 are nearly identical for the two non-equivalent monomers in the *P*3_1_21 crystals of unligated FKBP12.6, while in the X-ray structure of the E60Q variant of FKBP12 both of these phenyl rings move slightly inwards with the perpendicular reorientation of the Trp59 ring. Hence, in contrast to the steric compression which has been proposed to drive the flipping of the Trp59 ring when the interaction of residue 60 with the 50s loop is altered by mutation, the rotation of the Phe59 ring of FKBP12.6 appears to reflect a lack of strong interactions that enables this phenyl ring to occupy multiple conformations with similar energetics.

The differing orientation of the Phe59 ring in the two non-equivalent monomers of this crystal form can only arise from differences in lattice interactions. The highly similar backbone conformations for the non-equivalent monomers and the absence of any interactions with the neighboring lattice molecule that directly involve this phenyl ring suggest that there is only a modest difference in free energy between the parallel and perpendicular orientations of the Phe59 ring.

Of the 22 FKBP domains in the human genome, FKBP12.6 is the only one that contains a phenylalanine residue at the base of the active site (Galat, 2008 ▶). In addition to FKBP12, six other FKBP domains have a tryptophan residue at this position, while the other 14 FKBP domains contain either leucine, isoleucine or methionine at this site. The plasticity of the Phe59 ring in FKBP12.6 offers the potential for an active-site stereochemistry that could enable the design of binding selectivity with respect to the other FKBP domains.

### 3.3. Flipping of a peptide linkage at the tip of the 80s loop in FKBP12.6   

**Figure 3:**
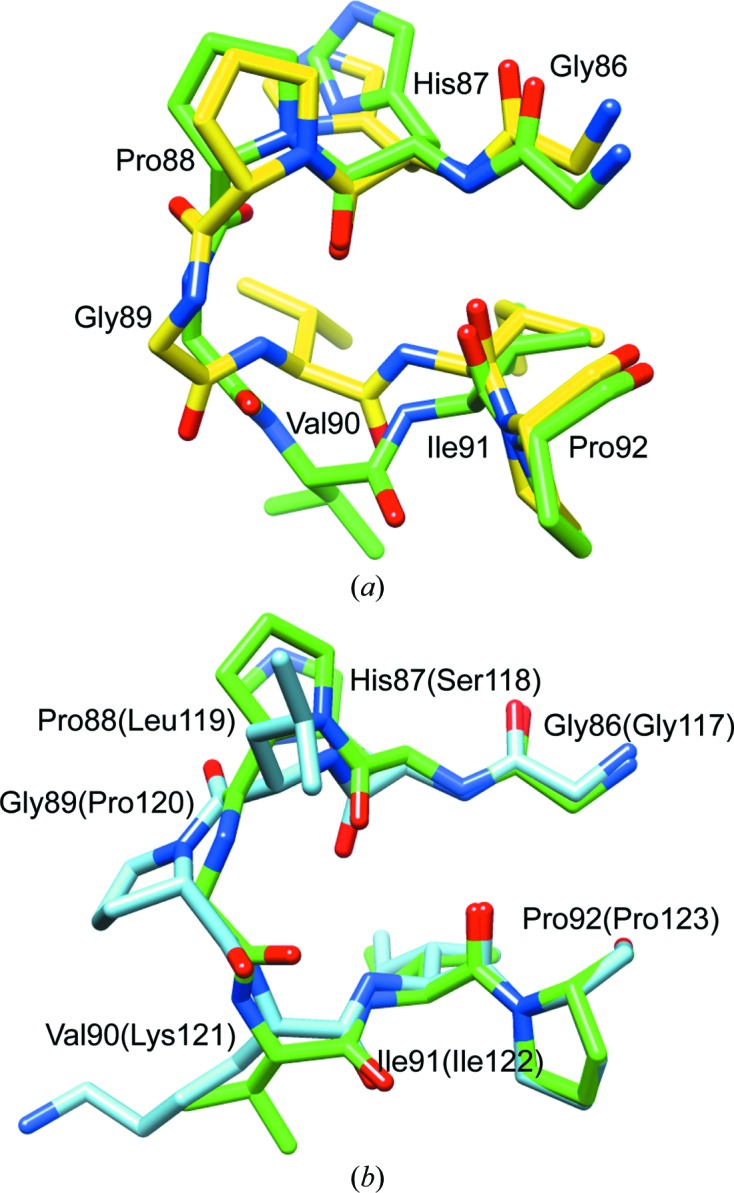
Superposition of the region surrounding the Gly89–Val90 peptide bond from the two crystal forms of FKBP12.6 and comparison with the corresponding segment of the first FKBP domain of FKBP51. The C atoms of the *P*2_1_ crystal form structure of cysteine-free FKBP12.6 are colored green, while those of the *P*3_1_21 crystal form structure are shown in yellow (*a*). The peptide linkage between Gly89 and Val90 is flipped in the *P*2_1_ crystal form compared with the *P*3_1_21 crystal form as well as in comparison to either the rapamycin-inhibited FKBP12.6 structure (Deivanayagam *et al.*, 2000 ▶) or the high-resolution apo FKBP12 structure (Szep *et al.*, 2009 ▶). In (*b*), this segment of the *P*2_1_ crystal form structure of cysteine-free FKBP12.6 is superimposed upon the homologous segment from the 1.00 Å resolution structure of the first FKBP domain of FKBP51 (PDB entry 3o5p; Bracher *et al.*, 2011 ▶), which is illustrated in blue.

Both non-equivalent monomers of the 1.70 Å resolution *P*2_1_ crystal form most notably differ from the 1.90 Å resolution *P*3_1_21 crystal form, as well as from rapamycin-inhibited FKBP12.6 and the various FKBP12 structures, by flipping of the peptide unit linking Gly89 and Val90 (Fig. 3 ▶ *a*). In the *P*2_1_ crystal form the backbone ψ torsion angle of Gly89 is −131°, compared with +2° in the *P*3_1_21 crystal form. Similarly, the backbone ϕ torsion angle of Val90 is −68° in the *P*2_1_ crystal form, compared with −128° in the *P*3_1_21 crystal form. In addition to reversing the directionality of the hydrogen-bonding interactions for this peptide linkage, the side chain of Val90 is significantly displaced. On the other hand, similarity in the backbone trace is recovered within a couple of residues on either side of the peptide-linkage flip.

In contrast to the flipping of the Phe59 phenyl ring considered above, the two conformations surrounding the Gly89–Val90 linkage are observed in two different crystal forms, so that compensation of the difference in free energy for these two conformational states in solution will reflect not only the differences in lattice interactions but also the differences in the crystallization conditions. Both FKBP12.6 crystallizations were carried out at high ionic strength and similar pH values. Regarding lattice interactions, none of the atoms in the Val90 side chain or in the peptide units on either side are within 5 Å of any atoms in the neighboring lattice molecule. These considerations provide credibility to the anticipation that the flipping of the Gly89–Val90 linkage may be significantly populated in solution.

Val90 lies at the tip of the 80s loop (also referred to as the polyproline loop), which connects the final two strands of the central β-sheet. This long loop provides a predominant proportion of the inter-protein interactions for each of the four protein–protein complexes that have been structurally determined for FKBP12 [*i.e.* calcineurin–FK506 (PDB entry 1tco; Griffith *et al.*, 1995 ▶), mTOR–rapamycin (PDB entry 2fap; Liang *et al.*, 1999 ▶), transforming growth factor β1 receptor (PDB entry 1b6c; Huse *et al.*, 1999 ▶) and bone morphogenetic protein receptor 1B (PDB entry 3mdy; Structural Genomics Consortium, unpublished work). Mutating Ile90 of FKBP12 to the valine found in the FKBP12.6 sequence reduces the binding affinity of the variant FKBP12 to calcineurin–FK506 by 100-­fold, while the analogous I90K substitution for the corresponding residue found in FKBP13, FKBP25 and the first FKBP domains of FKBP51 and FKBP52 results in a 2600-fold loss in binding affinity for calcineurin–FK506 (Futer *et al.*, 1995 ▶). The I90K mutation similarly disrupts the FKBP12–TGF β1 receptor complex (Chen *et al.*, 1997 ▶). The apparent conflict between the marked decrease in binding affinity for these homology-based point mutants of FKBP12 and the activities of the other FKBP domain proteins discussed above arises from the context-dependence of these sequence variations. In calcineurin-inhibition studies for FKBP12 and FKBP13 the swapping the sequence of an entire loop segment was found to have a markedly smaller effect on the inhibitory efficiency of the derived FKBP12 variant than did individual homologous mutations within this segment (Yang *et al.*, 1993 ▶). Despite the presence of a lysine side chain in the first FKBP domain of both FKBP51 and FKBP52 at the position corresponding to Ile/Val90, these proteins achieve high-affinity inhibition of mTOR by introducing additional intermolecular interactions *via* the 40s loop (März *et al.*, 2013 ▶).

It is interesting to note how closely the backbone of FKBP51 in the 80s loop segment follows that of the flipped peptide-linkage conformation which we have observed for FKBP12.6, despite substantial differences in sequence (residues 86–92 are GHPGVIP for FKBP12.6 and GSLPKIP for FKBP51). Not only do the terminal residues of this segment accurately superimpose (Fig. 3 ▶ *b*), the position and orientation of the peptide hydrogen-bond donors and acceptors throughout the loop are quite similar in the FKBP12.6 and FKBP51 (PDB entry 3o5p; Bracher *et al.*, 2011 ▶) structures. Likewise, the positioning of the side chain of residue 90 in this crystal form of FKBP12.6 closely follows that of this FKBP51 structure. The markedly different ϕ, ψ dihedral angles for Gly89 and Ile90 help to enable the backbone hydrogen-bond donors and acceptors that are shared in common between the two structures to adopt similar geometries despite the presence of the *cis*-proline linkage between Leu119 and Pro120 in the FKBP51 structure.

Owing to the recent efforts of Hausch and coworkers (Bracher *et al.*, 2011 ▶; Gopalakrishnan *et al.*, 2012 ▶; März *et al.*, 2013 ▶), there are 24 crystal structures with resolution limits of 2.0 Å or better for the first FKBP domain of FKBP51, either unligated, bound to FK506 or prospective lead compounds, or in a tertiary complex with the recognition domain of mTOR and rapamycin (PDB entries 3o5i, 3o5m and 4drk have two non-equivalent monomers in the asymmetric unit). As illustrated by unligated FKBP51 in six different crystal forms, the conformation at the tip of the 80s loop varies significantly for Leu119, Pro120 and Lys121 (Supplementary Fig. S3*a*). However, the structural variability is largely limited to these three residues (Supplementary Fig. S3*b*) and this variability appears to be primarily accounted for by a single mode of flexibility involving concerted rotations of the ψ angle of Ser118 and the ϕ angle of Lys121 (Supplementary Table S1). For all 24 high-resolution crystal structures, the variations in these two backbone torsion angles have r.m.s.d. values of 15.9 and 15.1°, respectively, and are correlated with an *r* value of −0.79. In contrast, the other ten backbone torsion angles from Gly117 to Pro123 have r.m.s.d. values that are on average threefold smaller.

The FKBP51 gene is believed to have arisen from an ancestral FKBP12 gene during early eukaryotic development (Galat, 2008 ▶, 2013 ▶). Given the partial functional overlap that is generally assumed among the various members of the FKBP domain family, it is of interest that the 80s loop of FKBP12.6, and presumably FKBP12, can readily access the basin of similar conformations occupied by FKBP51. In contrast, given the *cis*-peptide linkage at Pro120 in FKBP51 and the positive ϕ torsion angle of the homologous residue Gly89 in FKBP12 and FKBP12.6, it is unclear whether the FKBP51 structure can energetically access a conformation that resembles the major conformational state for these two proteins.

### 3.4. Spatial distribution of NMR resonance doubling for the backbone of FKBP12.6   

**Figure 4:**
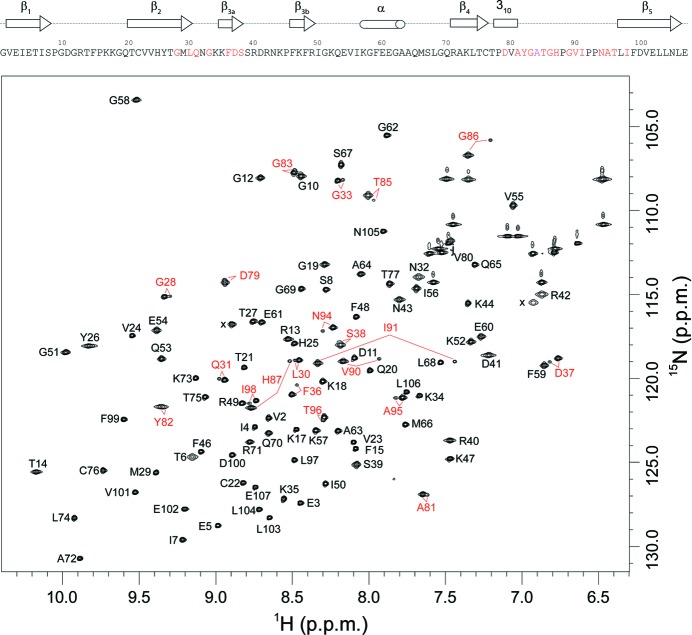
^1^H–^15^N two-dimensional NMR correlation spectrum of U-^2^H,^15^N-enriched wild-type FKBP12.6. Residues exhibiting resolved resonances for the minor slow-exchange conformation are indicated in red. Resonances were not observed in this spectrum for Ala84 and for Gly89 in the major slow-exchange state presumably owing to severe line-broadening arising from both rapid amide hydrogen exchange (Hernández *et al.*, 2009 ▶) and conformational exchange dynamics in the 80s loop (Mustafi *et al.*, 2013 ▶) as observed in the homologous FKBP12. The Gly89 resonance for the minor exchange state is observable at an approximately twofold lower contour level. The assessment of resonance doubling for Val80 is impeded by overlap with a side-chain amide resonance. Folded side-chain resonances are indicated with ‘x’.

Recently, we reported that FKBP12 exists as two distinct conformations which slowly interchange with a time constant of 3.0 s at 43°C (Mustafi *et al.*, 2013 ▶). The minor conformational state exists as 12% of the total population at 25°C and the residues for which the amide ^1^H–^15^N resonances exhibit doubling extend throughout much of the protein. Chemical shift analysis of the proline side-chain resonances demonstrated that all *X*–Pro peptide linkages of both the major and minor slow-exchange states are in *trans* configurations (Mustafi *et al.*, 2013 ▶), thus eliminating *cis*–*trans* prolyl isomerization as the structural basis for the slow conformational exchange. When we analyzed the analogous two-dimensional HSQC spectrum for U-^2^H,^15^N-enriched wild-type FKBP12.6, we observed extensive peak doubling for this protein as well (Fig. 4 ▶). The analogous two-dimensional HSQC spectrum for the cysteine-free variant of FKBP12.6 used in the crystallographic studies yielded the same pattern of peak doublings (Supplementary Fig. S4). As the NMR resonance assignment for this protein had not been reported, we carried out a set of triple-resonance experiments to assign the backbone resonances for both the major and minor forms. Averaging over the minor-state resonances that are well resolved and do not exhibit substantial line-broadening, the population of the minor state was estimated to be 5.1 ± 0.9%. A sample of U-^2^H,^13^C,^15^N-enriched FKBP12.6 provided sufficient sensitivity for the backbone assignment of the weak minor-state peaks, as illustrated for the segment Gly89–Ile91 (Supplementary Fig. S5).

**Figure 5:**
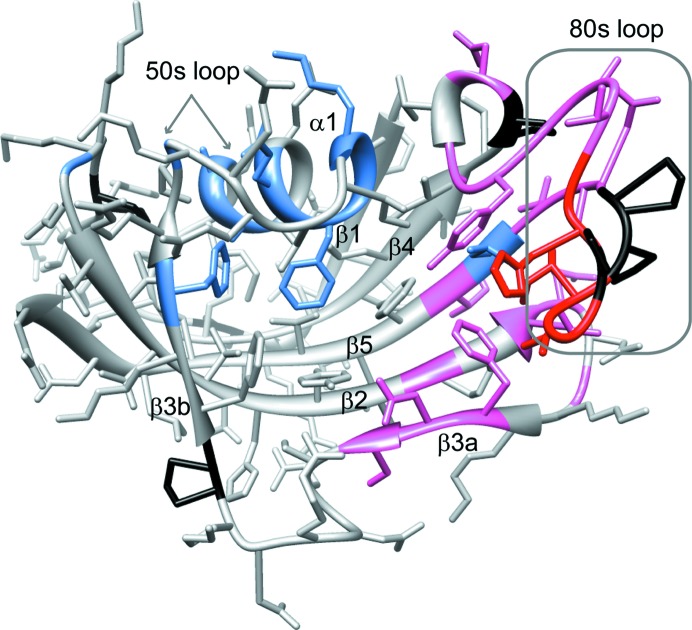
Structural distribution of residues exhibiting amide-resonance doubling owing to slow conformational exchange in FKBP12.6. Residues that yield doublings of their amide resonances separated by more than 0.15 p.p.m. (averaged as Δ^1^H and 0.2Δ^15^N; Garrett *et al.*, 1997 ▶) are indicated in red. Residues exhibiting smaller chemical shift differences between the two conformational states are indicated in pink. Residues that exhibit doubling in FKBP12 (Mustafi *et al.*, 2013 ▶) but not in FKBP12.6 are indicated in blue. Prolines are marked in black.

The residues exhibiting the largest differences in chemical shift between the doubled resonances of the major and minor states are in the region surrounding Gly89 and Val90 at the tip of the 80s loop (Fig. 4 ▶), similar to the pattern observed for FKBP12 (Mustafi *et al.*, 2013 ▶). In addition to the doubled resonances that occur within the 80s loop, the residues at the ends of strands β_2_, β_3_ and β_5_ nearest to the 80s loop also exhibited doubling (Fig. 5 ▶). No other amide-resonance doublings were observed. This behavior is in striking contrast to FKBP12, which not only exhibits a similar pattern of resonance doubling for the 80s loop and the adjacent strands of the β-sheet but also shows doubling for resonances in the α-­helix lining the opposite face of the active-site cleft and beyond into the beginning of the 50s loop (Mustafi *et al.*, 2013 ▶). Given that the perpendicular orientation of the Phe59 ring observed in molecule *A* of the *P*3_1_21 crystal form of FKBP12.6 appears to indicate a reduced level of conformational constraint imposed upon this side chain, the increased plasticity of this residue could serve to help to decouple the dynamics of the α-helix and the 50s loop region from the slow transition that is occurring in the region of the 80s loop.

Strong evidence that the spatially extensive set of resonance doublings for FKBP12 arise from a single collective slow conformational transition was drawn from the fact that introducing the H87V mutation suppressed all of these resonance doublings (Mustafi *et al.*, 2013 ▶). The population of the minor state was reduced at least 60-fold, despite the fact that the 1.70 Å resolution X-ray structure of this mutational variant (Mustafi *et al.*, 2013 ▶) is strikingly similar to that of the 0.92 Å resolution structure of the wild-type protein (Szep *et al.*, 2009 ▶). When the analogous H87V mutation was introduced into the cysteine-free FKBP12.6 background, a similar suppression of all resonance doubling was observed (Supplementary Fig. S6).

### 3.5. Exchange spectroscopy of the slow conformational transition in FKBP12.6   

**Figure 6:**
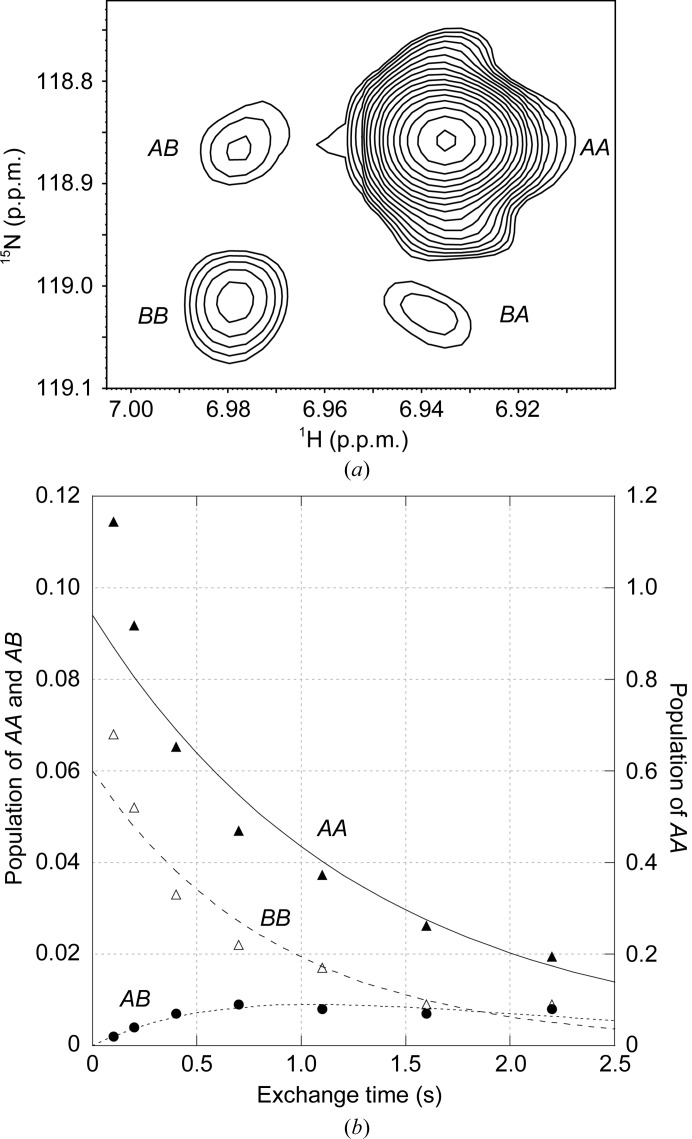
Kinetics of the slow conformational exchange in the C22V+C76I variant of FKBP12.6 at 43°C. (*a*) *zz*-exchange diagonal and cross-peaks of Asp37 in the major and minor conformational states at a mixing time of 1.1 s. (*b*) Time course for the peak intensities for the *AA* and *BB* diagonal peaks and the *AB* cross-peak.

If the rate of a two-state conformational transition is comparable to the ^1^H longitudinal NMR relaxation rates for the molecule, a two-dimensional *zz*-exchange experiment (Jeener *et al.*, 1979 ▶) can be used to determine the rate constant for the transition. For the analysis of cysteine-free FKBP12 (Mustafi *et al.*, 2013 ▶) the temperature was raised to 43°C in order to obtain sufficiently rapid conformational exchange dynamics. Similar conditions were applied to monitor the amide resonances of a U-^2^H,^15^N-enriched sample of cysteine-free FKBP12.6. The indirectly detected dimension of the *zz*-­exchange experiment can correspond to either the ^1^H or ^15^N frequency of the initially excited amide group. As illustrated for Asp37 in the C22V+C76I variant of FKBP12.6 at 43°C, the two diagonal peaks *AA* and *BB* arise from magnetization on ^1^H nuclei that remained in the same conformational state at both the beginning and the end of the exchange mixing period, while the two off-diagonal cross-peaks *AB* and *BA* arise from nuclei that change to the other conformational state during the mixing period (Fig. 6 ▶ *a*).

Following the analysis used for FKBP12 (Mustafi *et al.*, 2013 ▶), when the intensities of these peaks are plotted against each other as a function of the exchange mixing period (Fig. 6 ▶ *b*), an approximate exchange lifetime of 2.4 s is obtained by fitting the population of states *A* and *B*, their rate of conformational exchange and the average ^1^H *R*_1_ relaxation rate to a two-state model. Owing in part to the low population of the minor conformational state (5%), the cross-peak intensity never reaches 1% of the total initial peak signal. As a result, the quality of fit to the data is appreciably less robust than for the analogous measurements on FKBP12 (Mustafi *et al.*, 2013 ▶). Nevertheless, the conformational transition lifetime obtained for FKBP12.6 is reasonably close to the 3.0 s obtained for FKBP12. Assuming the activation energy of 70 kJ mol^−1^ that was determined for FKBP12 (Mustafi *et al.*, 2013 ▶), the conformational transition lifetime for FKBP12.6 at 25°C would be ∼15–20 s.

## 4. Conclusion   

Previous crystallographic analyses of FKBP12 have concluded that alterations in the hydrogen-bonding interactions of the backbone at the start of the 50s loop can give rise to a reorientation of the relatively distant active-site Trp59 ring. The resultant occlusion of the standard ligand-binding site were argued to be driven by compression forces that impinge upon this aromatic ring (Szep *et al.*, 2009 ▶). In the *P*3_1_21 crystal form of unligated FKBP12.6, a similar perpendicular reorientation of the Phe59 ring is observed. However, in this case the absence of substantial steric interactions appears to allow the facile sampling of the two orientations of the Phe59 ring in the same crystal form. This enhanced plasticity in the active site of FKBP12.6 is likely to contribute to the marked attenuation in the spatial extent of the residues that exhibit doubling of their amide resonances compared with those of the homologous FKBP12.

The *P*2_1_ crystal form of FKBP12.6 exhibits a flipping of the peptide group linking Gly89 and Val90 which, given the absence of nearby lattice interactions, would appear to be a relatively unconstrained transition. This novel conformation for the tip of the 80s loop of FKBP12.6 yields a set of backbone hydrogen-bonding interactions that closely mimic those of the analogous protein-recognition site in the first FKBP domain of FKBP51. As noted in the recent crystallographic analysis of the complexes formed between both FKBP51 and FKBP52 with the recognition domain of mTOR (März *et al.*, 2013 ▶), plasticity in the 80s (and 40s) loops of these domains enable selective functional recognition that appeared to be unfeasible based on the earlier crystal structures of the FKBP12–mTOR complex and of the isolated FKBP domains of FKBP51 and FKBP52. Unanticipated structural mimicry, such as that illustrated by the flipping of the peptide group observed in FKBP12.6, may help to provide insight into the still poorly understood complementarity among the physiological roles of the various members of the FKBP domain proteins.

## Supplementary Material

PDB reference: FKBP12.6, 4iq2

PDB reference: 4iqc

Supporting Information.. DOI: 10.1107/S1399004713032112/kw5080sup1.pdf

## Figures and Tables

**Table 1:** Data-collection, refinement and model details

	Condition 1	Condition 2
Data collection		
Space group	*P*2_1_	*P*3_1_21
Resolution range ()	39.21.7 (1.761.70)	45.71.9 (1.931.90)
No. of unique reflections	20138	20089
Average multiplicity	3.5 (2.7)	8.8 (4.2)
Completeness (%)	95.8 (77.7)	99.3 (99.5)
Mean *I*/(*I*)	4.6 (1.2)	22.5 (0.9)
*R*_merge_ †	0.137 (0.51)	0.094 (0.894)
Refinement		
Resolution limits ()	39.21.7	45.71.9
No. of reflections	20126	20089
*R*_work_	0.226	0.195
*R*_free_	0.251	0.222
No. of non-H atoms		
Protein	1644	1644
Malonic acid	1	
Water	241	111
Average *B* (^2^)		
Wilson	16	31
All atoms	22	35
Solvent	29	41
R.m.s. deviations from ideal values‡		
Bond lengths ()	0.01	0.01
Bond angles ()	1.11	1.08
Ramachandran plot§		
Favored (%)	97.6	97.1
Allowed (%)	2.4	2.9
Disallowed (%)	0	0
Rotamer outliers (%)	0.6	0
Clashscore	5.3	8.1

† *R*_merge_ = 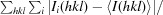 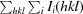, where *I*_*i*_(*hkl*) is the *i*th observation of reflection *hkl*, while *I*(*hkl*) is its mean intensity.

‡ Engh Huber (1991 ▶).

§ Chen *et al.* (2010 ▶).

## Footnotes

1 Supporting information has been deposited in the IUCr electronic archive (Reference: KW5080).
