# Supplementary material for: Crystal structure and conformational flexibility of the unligated FK506-binding protein FKBP12.6
Source: Acta Crystallogr D Biol Crystallogr. 2014 Feb 15;70(Pt 3):636–46. doi: 10.1107/S1399004713032112 (PMC3949516; doi:10.1107/S1399004713032112)
Supplement: Supplementary file 1 [file d-70-00636-sup1.pdf]

Supporting Information

Crystal structure and conformational flexibility of the unligated FK506-binding protein FKBP12.6

Hui Chen[a‡], Sourajit M. Mustafi[a], David M. LeMaster[a,b], Zhong Li[a], Annie Héroux[c], Hongmin Li[a,b] and Griselda Hernández[a,b]*

[a]Wadsworth Center, New York State Department of Health Empire State Plaza, Albany, New York, 12201, USA [b]Department of Biomedical Sciences, School of Public Health, University at Albany - SUNY, Empire State Plaza, Albany, New York, 12201, USA [c] Department of Biology, Brookhaven National Laboratory, Upton, New York 11973,USA

[‡] Present address: Life Sciences Institute, University of Michigan, Ann Arbor, Michigan 48109, USA

Correspondence may be addressed to Griselda Hernández, Wadsworth Center, New York State Department of Health, Empire State Plaza, Albany, New York, 12201, USA. Telephone: (518)474-4673, Fax: (518)473-2900, E-mail: gch02@health.state.ny.us

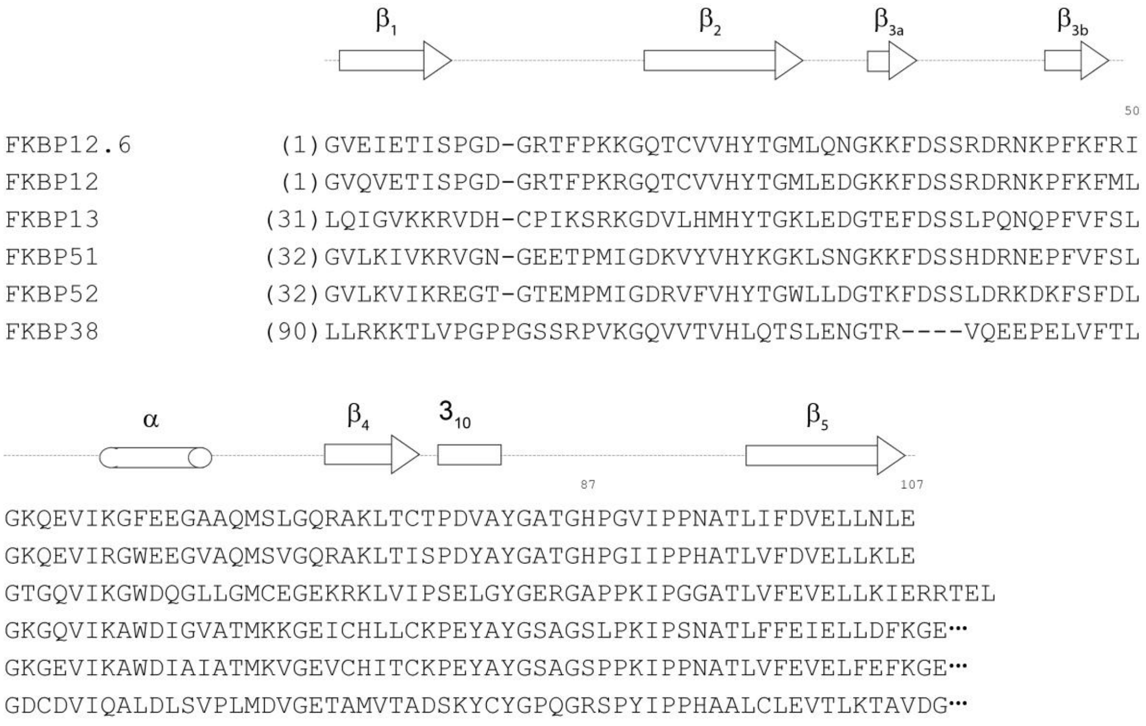

Figure S1 Sequence alignment for the human FKBP domains of FKBP12.6, FKBP12, FKBP13, FKBP51, FKBP52 and FKBP38 (first domain for FKBP51 and FKBP52).

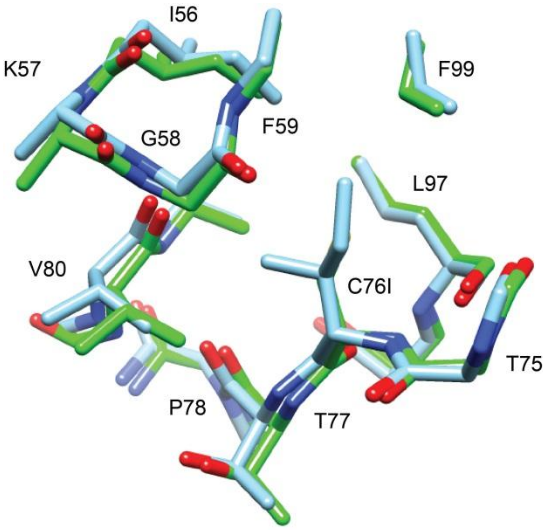

Figure S2 Superposition of region surrounding the C76I substitution from the 1.90 Å resolution structure of the cysteine-free variant of FKBP12.6 and the 1.70 Å resolution structure rapamycin-inhibited FKBP12.6. The carbons of the rapamycin-inhibited structure of FKBP12.6 are colored in green while those of the unligated cysteine-free variant are given in light blue. The Cys 76 S^{γ} in the rapamycin-inhibited structure of FKBP12.6 (Deivanayagam *et al.*, 2000) precisely superimposes on the Ile 76 $C^{\gamma 1}$ from the cysteine-free variant. The Ile 76 $C^{\gamma 2}$ contacts the sidechain and C^{α} of Val 80, causing a displacement of C^{α} of less than 0.7 Å.

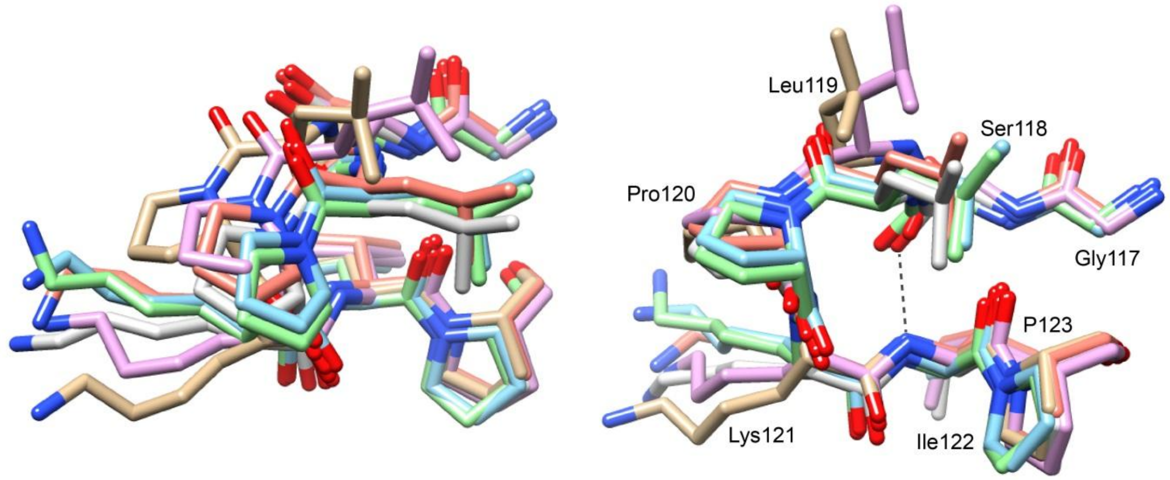

Figure S3 Superposition of the tip of the 80's loop for the first FKBP domain of FKBP51in the unligated state for six different crystal forms of 2.0 Å resolution or better, viewed head-on (a) and rotated ~90° (b). Following superpositioning of the complete domains, the structures represented are PDB codes 3O5E (magneta), 3O5F (blue), 3O5L (gray), 3O5M (rust), 3O5O (green) and 3O5P (gold). Despite an appreciable range of conformations exhibited by residues 119 to 121, the positioning of the hydrogen bond between the carbonyl oxygen of Ser 118 and the amide of Ile 122 is well preserved as are the connected backbone segments.

Table S1 Mean and standard deviation for the backbone torsion angles of the tip of the 80's loop among the 24 crystal structures of the first FKBP domain of FKBP51with 2.0 Å resolution or better.

	ϕ_{ave}	σ	ψ_{ave}	σ
Gly 117	68	3.4	-165	3.4
Ser 118	-146	6.2	90	15.9
Leu 119	-71	5.0	147	4.9
Pro 120	-88	5.6	-12	6.1
Lys 121	-82	15.1	-32	8.8
Ile 122	-115	8.2	115	2.4
Pro 123	-74	3.9	170	3.1

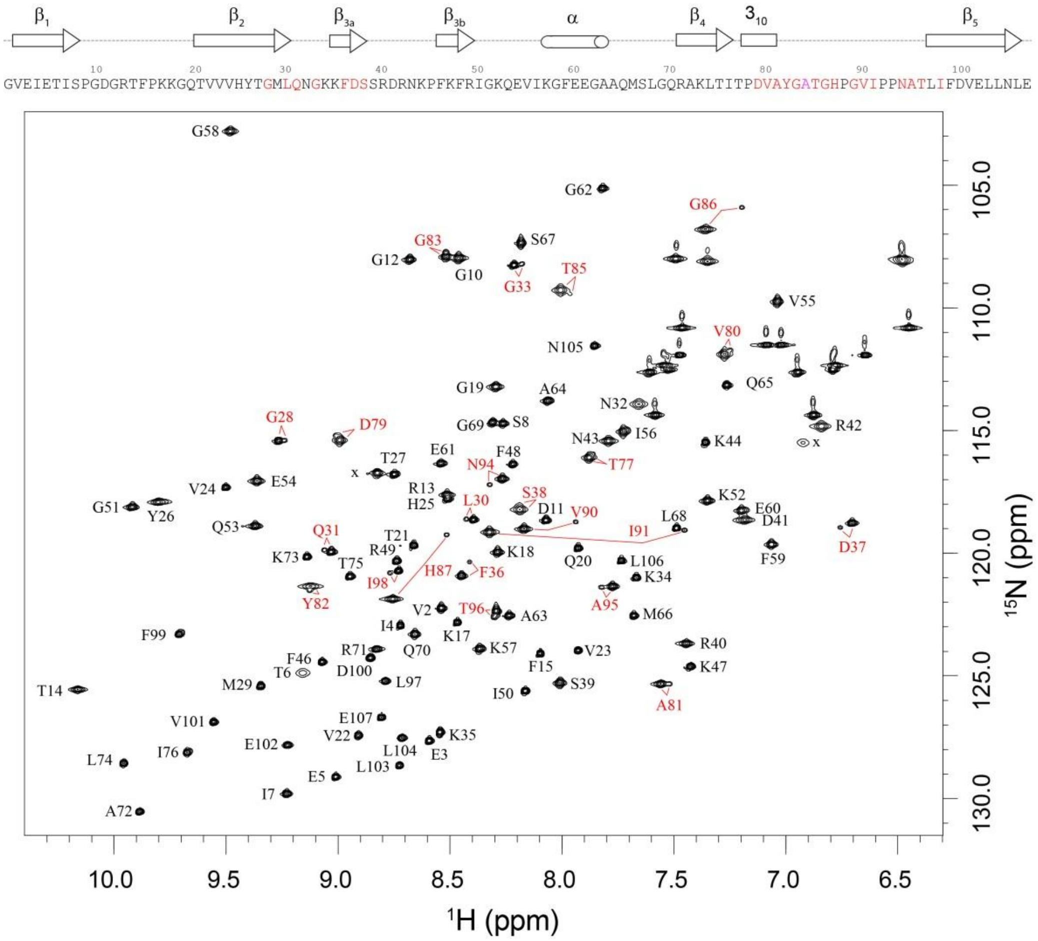

Figure S4 ^{1}H-^{15}N 2D NMR correlation spectrum of U-^{2}H,^{15}N enriched C22V,C76I variant of FKBP12.6. Residues exhibiting resolved resonances for the minor slow exchange conformation are indicated in red. Resonances were not observed in this spectrum for Ala 84 and for Gly 89 in the major slow exchange state presumably due to severe linebroadening arising from both rapid amide hydrogen exchange (Hernández *et al.*, 2009) and conformational exchange dynamics in the 80's loop (Mustafi *et al.*, 2013) as observed in the homologous FKBP12. The Gly 89 resonance for the minor exchange state is observable at a ~2-fold lower contour level. Folded sidechain resonances are indicated with 'x'.

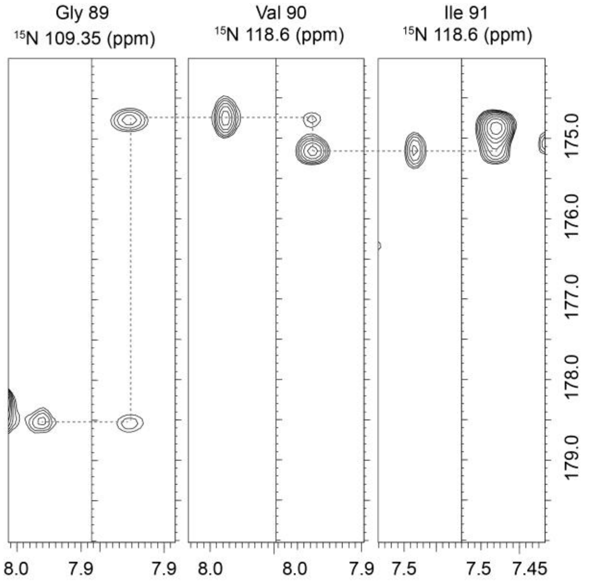

Figure S5 Backbone resonance assignment of the minor slow exchange conformation of human FKBP12.6. Strips from the HNCO (left) and HN(CA)CO (right) data sets at the ^{15}N frequencies for the amide resonances of the minor slow exchange conformation are illustrated for residues Gly 89 to Ile 91.

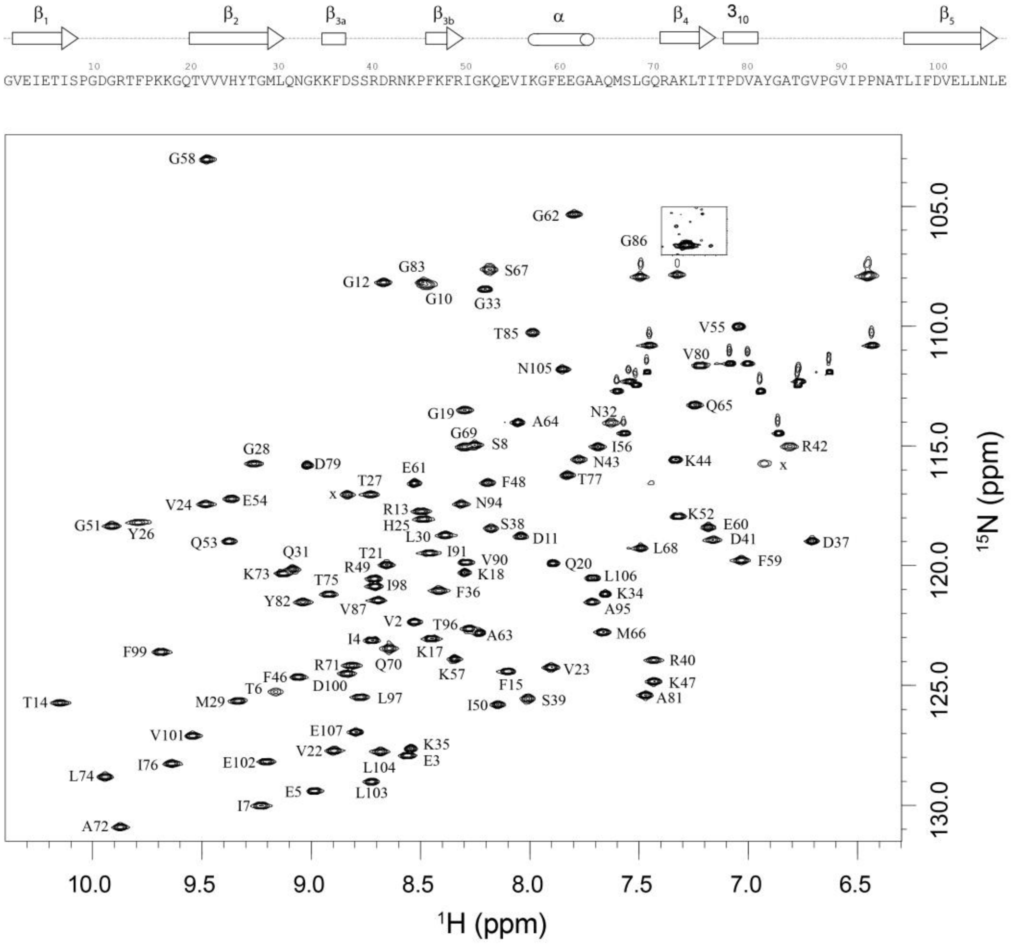

Figure S6 ^{1}H-^{15}N 2D NMR correlation spectrum of U-^{2}H,^{15}N enriched H87V variant of cysteine-free FKBP12.6. The spectral region surrounding the resonance for Gly 86 is plotted at an 8-fold lower contour to illustrate the absence of a minor peak arising from slow conformational exchange. Folded sidechain resonances are indicated with ‘x’.

REFERENCES

Deivanayagam, C. C., Carson, M., Thotakura, A., Narayana, S. V., & Chodavarapu, R. S. (2000). *Acta Cryst. D*, **56**, 266-271.

Hernández, G., Anderson, J. S., & LeMaster, D. M. (2009). *Biochemistry*, **48**, 6482-6494.

Mustafi, S. M., Chen, H., Li, H., LeMaster, D. M., & Hernández, G. (2013). *Biochem. J.*, **453**, 371-380.
